# Intrinsic Reward Modulates Word Learning in Both Oral and Written Contexts

**DOI:** 10.5334/joc.499

**Published:** 2026-04-30

**Authors:** Haniya Zaka, Samuel Evans, Pablo Ripollés, Saloni Krishnan

**Affiliations:** 1 Department of Psychology, Royal Holloway, University of London, UK; 2 Division of Psychology and Language Sciences, UCL, London, UK; 3 Department of Psychology, New York University, New York, NY 10003, US; 4 Music and Audio Research Lab (MARL), New York University, New York, NY 10003, US; 5 Center for Language, Music and Emotion (CLaME), New York University, Max-Planck Institute, New York, NY 10003, US; 6 Professor, Department of Psychology, Royal Holloway, University of London, UK

**Keywords:** vocabulary, reward, pleasure, long-term memory, contextual word learning

## Abstract

Previous studies show that word learning from context can be intrinsically rewarding, even in the absence of external feedback or incentives. Intrinsic reward activates the brain’s reward-memory circuit, leading to enhanced memory for words people enjoyed learning. Existing studies have tested the link between contextual word learning and reward in the written domain (i.e., through reading). However, word learning from context often occurs in the oral domain, where conversations provide a rich source for acquiring vocabulary in both first and second language learning. In this online behavioral study, we investigate whether word learning triggers enjoyment across modalities, focusing on listening, reading, and listening and reading in combination. In all the modalities, we found that when words are successfully learned from context, people report greater levels of enjoyment. We also found that words that were associated with higher enjoyment during learning were recognised more accurately than those associated with lower enjoyment 24 hours later. Our results demonstrate the relevance of intrinsic reward for language learning and suggest that the link between contextual word learning and reward operates on higher-level word representations, rather than on modality-specific ones.

We continually encounter unfamiliar words in written text, such as newspaper articles and books, as well as in the oral domain, such as in audiobooks, podcasts, and in conversation. We extract and refine our understanding of these new word meanings from the context in which they appear, growing our vocabulary in the process (Nagy et al., 1985; Swanborn & De Glopper, 1999). *Why* do we engage in such learning? Recent empirical studies probing incidental word learning in written contexts, using behavioural, physiological, neuroimaging and pharmacological approaches, indicate that we find the process of extracting word meaning from context intrinsically rewarding (Ripollés et al., 2014, 2016, 2018). This fits with theoretical accounts postulating that a link between the brain’s reward and language systems may have been crucial for the evolution of human language (Syal & Finlay, 2011). Despite that fact that we learn new words from both oral and written contexts, the relationship between reward and contextual word learning has exclusively been studied using written stimuli. If intrinsic reward plays a general role in language learning, it should be observed in spoken language contexts.

This gap is particularly striking given the central role of spoken language in vocabulary acquisition. Children (including children with DLD: Developmental Language Disorder) learn new words in oral contexts (e.g., through being read stories; Flack et al., 2018; Horst et al., 2011; Rohlfing et al., 2018). Adults also acquire new words in a foreign language in oral contexts, such as by participating in conversations and engaging with oral content in stories, films, television, and podcasts (Bisson et al., 2021; R. Brown et al., 2008; Elley, 1989; Lenhart et al., 2018; Perez et al., 2018; Vidal, 2011). Research also shows that word learning from oral contexts is a valuable source of vocabulary for adults engaged in second language learning (Verga & Kotz, 2013). Finding a relationship between reward and word learning using auditory stimuli would indicate this link operates on higher-level, modality-independent representations of words. In this study, we fill this gap in the literature and investigate whether the relationship between reward and successful word learning from context is observed across both oral and written language modalities.

## Using a contextual word learning paradigm to study intrinsic reward

In this study, we use a contextual word learning paradigm, which closely mimics real-life vocabulary learning (Mestres-Missé et al., 2007). Participants decipher a new word’s meaning from the context provided by two sentences. For instance, when encountering the novel pseudoword *jedin* in these sentences, “Every Sunday the grandmother visited the *jedin*” and “The man was buried in the *jedin*”, readers may infer that *jedin* refers to a graveyard or cemetery. In everyday life, many words are learned incidentally in this manner, through exposure to meaningful contexts rather than direct instruction (Akhtar, 2004; Henderson et al., 2015), and without any need for explicit feedback.

Our use of a contextual word learning paradigm to investigate reward is supported by the growing body of evidence indicates that successfully mapping a new word form to meaning under these conditions is intrinsically rewarding (Angwin et al., 2019; Bains et al., 2024; Ripollés et al., 2014, 2016, 2018). In these studies, participants provided subjective ratings of enjoyment immediately after encountering novel words embedded in a pair of sentences. Enjoyment (provided during the encoding session) was consistently higher for words whose meanings were successfully inferred than for those that were not learned (Angwin et al., 2019; Bains et al., 2024; Ripollés et al., 2014, 2016, 2018). These effects have been replicated in adults with diverse language backgrounds, including Spanish, English, and German (Angwin et al., 2019; Ripollés et al., 2016; Vavra et al., 2023), as well as at different developmental stages, such as in children aged 10–18 years (Bains et al., 2024). This paradigm is particularly well suited to isolating intrinsic reward from confounding factors such as attention and novelty. Prior work contrasts congruent sentence pairs, in which a coherent meaning for the novel word can be inferred, with incongruent pairs, in which the two sentences evoke incompatible meanings and learning cannot occur (Mestres-Missé et al., 2007; Ripollés et al., 2016, 2018). Although participants can successfully detect incongruency, increased pleasure is observed only for congruent trials and not for incongruent trials, even when focusing on successful trials alone (Ripollés et al., 2016, 2018). Further, these effects do not extend to arousal ratings, indicating that subjective enjoyment is not a proxy for attention or fatigue (Ripollés et al., 2016).

Converging evidence from neuroimaging and pharmacological studies further implicates reward processing systems in contextual word learning. In an fMRI study, brain activity in reward and dopaminergic related regions such as the ventral striatum was greater during encoding for words that were correctly learned as compared to those which were not learned, or for incongruent sentences (Ripollés et al., 2014). Providing causal evidence for the involvement of the reward and dopaminergic system in this form of learning, the administration of a dopaminergic precursor (levodopa) and a dopaminergic antagonist (risperidone) ingested before learning, increased and decreased, respectively, both the learning rate and the enjoyment experienced by the participants in this contextual word learning paradigm (Ripollés et al., 2018).

## The link between reward and memory

Increased reward has been consistently linked to improvements in long-term memory (Adcock et al., 2006; Dickerson & Adcock, 2018; Murayama & Kitagami, 2014). In a seminal study, participants were more likely to recognise scenes presented after high-reward cues relative to low reward cues (Adcock et al., 2006). High-reward cues were also associated with activation in core reward, dopaminergic, and memory related regions, including the ventral tegmental area, ventral striatum, and hippocampus (Adcock et al., 2006). Mechanistically, these effects are thought to reflect the role of dopamine in mediating long-term potentiation processes via a memory loop linking the hippocampus, the ventral striatum and the dopaminergic midbrain (i.e., the ventral tegmental area and the substantia nigra; the SN/VTA-Hippocampal loop; Lisman et al., 2011; Lisman & Grace, 2005; Shohamy & Adcock, 2010).

Importantly, studies indicate that *intrinsic* reward-related signals also facilitate the entrance of items into long-term memory (e.g., music reward; Ferreri et al., 2021; curiosity, Garvin & Krishnan, 2022; Gruber et al., 2014; Kang et al., 2009; Marvin & Shohamy, 2016). Using the contextual word learning paradigm described above, Ripollés and colleagues (2016) demonstrated that subjective ratings of enjoyment during learning were higher for words that were successfully remembered 24 hours after learning. At the neural level, successful long-term retention was associated with brain activity and functional connectivity in the SN/VTA-Hippocampal loop (Ripollés et al., 2016). Crucially, these effects were not observed for novel words embedded in incongruent sentence contexts, even when such items were correctly identified as incongruent during learning and subsequently recognised as such in a memory test (Ripollés et al., 2016). Providing further causal evidence for a role for dopaminergic reward systems in learning, ingesting a dopaminergic precursor (levodopa) and a dopaminergic antagonist (risperidone) before learning, increased and decreased, respectively, the number of words retained after a 24-hour consolidation period (Ripollés et al., 2018).

## Studying intrinsic reward across modality

All the aforementioned studies have probed the link between reward and contextual word learning using written stimuli. Observing this relationship in the oral domain would open new lines of investigation, allowing for the study of the link between reward and language processing in younger children who do not read fluently, adults who are illiterate, or individuals who struggle to read. We hypothesise that links between reward processing and semantics operate on higher-level language representations and are independent of modality (oral/written). We consequently postulate that the facilitatory link between reward and language learning will also be observed in the oral domain, given the importance of this modality for real-world word learning in both children and adults (Bisson et al., 2021; Flack et al., 2018). We also predict that the relationship between reward and learning will be of a similar strength across modality. However, it is important to emphasise a different prediction could be made. Orthography is less transient than phonology and less variable across individuals and contexts. Skilled readers can make use of orthographic input to reduce processing and memory demands involved in listening, thereby supporting learning of new word forms and their meaning. From this perspective, the processing and memory demands of a listening task could diminish the subjective experience of reward and constrain the engagement of reward systems in supporting later memory. In this vein, we also included a final condition, reading and listening, somewhat akin to providing subtitles on videos. This was because learning is enhanced when both reading and listening contexts are jointly provided (Valentini et al., 2018). If processing demands impact subjective reward, then participants should experience the greatest reward in this condition.

In this online behavioral study, we investigate the relationship between reward -measured using ratings of enjoyment- and contextual word learning in three modalities: reading, listening, or reading and listening. Our primary hypothesis was that enjoyment ratings would be higher when participants successfully extracted a word’s meaning compared to when they were unsuccessful or unable to infer a meaning, across all three modalities: reading, listening, and reading-while-listening. We also hypothesised that across all three modalities, subjective enjoyment (during learning) would be higher for words that were successfully recognised after a 24-hour delay, compared to enjoyment for words that were not recognised.

## Methods

### Ethics

This study received approval from the Central Ethics Committee at Royal Holloway, University of London. All participants provided written informed consent.

### Power analysis

Prior to starting the experiment, we determined sample size using ANOVAs. We performed a sample size analysis using MorePower (Campbell & Thompson, 2012), which showed that we needed a minimum of 28 participants in each modality to detect the key interaction of congruency (M+/M–, see detailed methods below) and accuracy (correct/incorrect) on enjoyment ratings across the 3 groups (Listening, Reading, Listening + Reading), taking into account an effect size of η^2^ = 0.14 with 90% power. We chose to use mixed-effects modelling to analyse our data, as this allows us to account for variability within and across participants and items simultaneously, while also handling unbalanced designs and missing data well. We have therefore conducted post-hoc simulations of power using mixed effects models (see Appendix 1). These analyses suggest we had over 90% sensitivity to detect effects of congruency, accuracy and the interaction between congruency and accuracy. With 110 participants, these post-hoc analyses suggested we also had >80% sensitivity to detect a three-way interaction between congruency, accuracy and modality, assuming a fixed effect of 0.3.

### Participants

Participants were recruited online for an undergraduate student project through the university SONA pool as well as through word-of-mouth advertisement. Participants were offered the chance to win a £50 Amazon voucher. Our inclusion criteria were being a native English-speaking adult between the ages of 18–40 with normal or corrected-to-normal vision. Exclusionary criteria were the presence of a history of any known neurological disorder, developmental disorders, or speech, language, or hearing disorders. Participants who did not complete the experiment had their data removed and were replaced by other participants. Participants were assigned at random to one of the three modalities: reading, listening, or reading + listening. Given our power analyses, we aimed to recruit 90 participants (30 per group, to account for attrition) between the ages of 18–40. We stopped data collection when we achieved N = 30+ in each group; because of random assignment to group our final sample size was 112 (Listening N = 36, Reading N = 42, Listening and Reading N = 34).

To assess whether there were any demographic differences between the 3 groups, we used JASP version 0.19.1.0 (Love et al., 2019) to calculate Bayes factors using default priors (BF_01_, reflecting how likely data are to support the null hypothesis; Wagenmakers et al., 2018). For instance, a BF_01_ = 3 indicates that the data are three times more likely under the null than under the alternative hypothesis (Lee & Wagenmakers, 2014). For continuous variables we used Bayesian one-way ANOVAs. For categorical variables, we used Bayesian Contingency Tables with independent multinomial sampling (Jamil et al., 2017). The groups were comparable in terms of age (BF_01_ = 7.3; Listening: 22.56 ± 4.31 years; Reading: 23.08 ± 5.38 years; Listening and Reading: 21.85 ± 4.87 years), gender (BF_01_ = 5.34; Listening: 28 women; Reading: 32 women; Listening and Reading: 31 women), and sensitivity to reward (as measured with the BIS/BAS; Vervoort et al., 2015; BIS: BF_01_ = 8.40, Listening: 21.8 ± 3.77; Reading: 21.54 ± 4.41; Listening and Reading: 22.38 ± 3.56; BAS: BF_01_ = 3.5, Listening: 34.11 ± 5.39; Reading: 34.28 ± 5.17; Listening and Reading: 32.29 ± 5.58).

### Stimuli

The stimuli comprised 40 pairs of sentences ending in a novel pseudoword. The pseudoword stood in for a noun. All pseudowords respected the phonotactic rules of English, were between 1–2 syllables, 5–7 letters in length, and were generated using Wuggy (Keuleers & Brysbaert, 2010). The sentences were developed for a previous study and slightly adapted and validated for British speakers (for further details, see Bains et al., 2024). The average cloze probability for first sentences was 20.34% while for second sentences it was 79.26% (see Appendix 2 for further details). Auditory versions of these stimuli were recorded by a native southern British-English speaker using audacity and a Rode NT1 A microphone with a Focusrite Scarlett 2i2 USB audio interface. Recordings were made at 44,100 Hz with 32bit quantization in a quiet room using a Marantz Professional Tabletop Sound Shield. The average length of the recorded sentences was 2.20 ± 0.29 seconds.

During the experiment, in half of the sentence pairs, the meaning evoked by the pseudoword was congruent and, therefore, it was possible to extract the meaning of the new word (M+ condition; e.g., sentence 1: “Few countries are now ruled by a cyche”; sentence 2: “In the palace lives the king and the cyche”. Cyche means *queen* and was congruent with both the first and second sentence). For the other half of the sentence pairs, the second sentences were randomized so that they no longer matched their original first sentences. In this case, the new-word could not be correctly associated with a congruent meaning across the sentences (incongruent or M– condition; e.g., sentence 1: “She hit the mouse with a *muden*.” – broom was one possible meaning of *muden*. Sentence 2: “Anna had lots of long beautiful *muden*”- Hair was now another possible meaning of *muden*, but this was not congruent with the first sentence). A correct response for the M– condition involved the participant indicating that the sentences did not provide a congruent meaning. While participants might incorrectly associate a meaning to a M– new word (e.g., the one evoked by the second sentence), this response is considered incorrect. Sentence assignment to M+ and M– conditions was counterbalanced. In other words, the 20 pairs of sentences that served as M+ in one version of the experiment were part of the M– condition in the other version.

### Design

After providing informed consent, participants were told that they would be exposed to new words that they were expected to learn. Before they started the learning phase, they were informed that they would be tested on their learning performance on the following day.

#### Contextual word learning task

**Figure 1:**
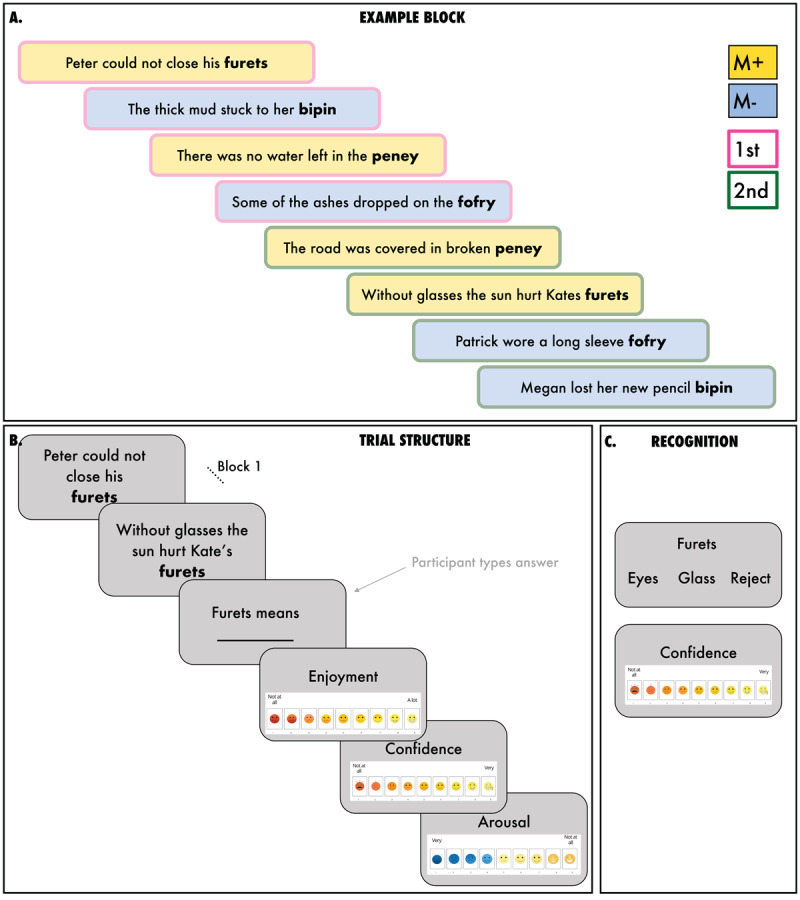
**A.** Schematic overview of block structure, illustrating the sentence congruency manipulation (congruent: M+, yellow; incongruent: M–, blue). Each block comprised 2 pairs of M+ sentences and 2 pairs of M– sentences presented randomly. Participants were presented with the ‘first’ sentence from all sentence pairs (pink outlines), before encountering the second sentence of the pair (green outlines). **B.** Schematic illustration of the trial structure. **C.** Schematic outline of the recognition trial.

During the learning phase on Day 1, participants encountered 40 trials. Trials were presented in blocks of four sentence pairs (see Figure 1 for schematic). Each block comprised 2 pairs of M+ sentences and 2 pairs of M– sentences presented in a random order. For the Reading condition, participants were shown a screen with the first sentence from a sentence pair; this stayed onscreen for 5s. They then saw three more “first” sentences from three other sentence pairs. Once all first sentences were presented, participants were shown the second sentences in a random order. The first and second sentences were presented separately, following previous validated methodology (Ripollés et al., 2014, 2016, 2018), to increase task difficulty and better simulate real-life word learning from context. In natural settings, individuals typically encounter unfamiliar words in sentences that are spaced out in time. After encountering the second sentence of a pair, which also remained onscreen for 5s, they were prompted to enter the meaning of the pseudoword or type the word “reject”, which would indicate that they believed the two sentences did not have a congruent meaning.

After they typed an answer, participants rated their enjoyment, confidence and arousal (confidence is the focus of a different study) using 9-point Likert visual scales ranging from 1–9 (shown in Figure 1). Specifically, they answered the following three questions, “How much did you enjoy that?”, “How confident do you feel in your answer?”, and “How tired are you feeling right now?”. In this work, our main focus is on enjoyment (i.e., reward).

#### Modality manipulations

The Listening and Listening + Reading conditions followed the same general paradigm as the Reading condition, with minor adjustments reflecting modality differences. In the Listening condition, the sentence was presented aurally without any accompanying visual text. Mimicking the presentation of the other two conditions, the stimulus was presented within a 5s window after which they continued to the next trial in the Listening condition. As the sentences were ~2.20 ms in length, participants experienced a short silence after auditory stimulus presentation in the Listening condition. For the Listening + Reading condition, the sentence was presented aurally at the same time as it was shown visually.

#### Recognition task

A recognition test followed a consolidation period of at least 24 hours. Regardless of the modality they had completed, participants encountered the pseudowords from the previous day presented both aurally and visually (for both M+ and M– conditions). They were then provided with three options (two meanings and the option to reject; as in Ripollés et al., 2014, 2016, 2018). For the M+ condition, these included the real meaning of the word (correct), a meaning consistent with another pair of sentences presented during the experiment (incorrect), and an option to reject (i.e., to incorrectly identify the trial as a non-congruent M– trial in which meaning cannot be extracted). For the M– condition the options were the meaning evoked by the second sentence (incorrect), the meaning consistent with another pair of sentences presented during the experiment (incorrect), and an option to reject (i.e., to correctly identify the trial as a non-congruent, M– trial in which meaning cannot be extracted correctly). After providing a response on Day 2, participants rated their confidence in their memory using a 9-point Likert visual scale.

### Procedure

Participants completed the experiment online using the Gorilla platform www.gorilla.sc (Anwyl-Irvine et al., 2020). After giving informed consent and completing a short demographics questionnaire, all participants completed a quick sound check to ensure that they had functioning headphones or speakers (they were played a word and had to type it in). Participants had to pass this test to continue. They were then automatically randomly assigned to a modality: reading, listening, or reading and listening.

Participants were given detailed instructions to ensure that they understood the task objectives, i.e., that they had to learn the words and they would be tested on their memory the next day. Critically, they were told the meaning should make sense in both the sentences the word appeared in. They were also told that in some trials, a correct answer involves reporting incongruency, i.e., that no reasonable meaning could be inferred. Participants then completed six practice trials. During the practice, they encountered 3 words embedded in M+ sentences, and 3 words embedded in M– sentences. They received feedback on their performance, as well as being shown the correct answer. This practice session ensured that participants understood the requirements of the task, and how to answer for both M+ and M– trials.

After this, participants completed the task without any feedback (i.e., at no point were they told whether their answers were correct or incorrect). Finally, participants were asked to fill in the Behavioral Inhibition System/Behavioral Approach System (BIS/BAS) scales, which assessed their sensitivity to reward (Vervoort et al., 2015). At the end of the task, participants were prompted to enter their email addresses, to continue the second part of the experiment the next day.

Twenty-four hours later, they were emailed a link to the second part of the experiment. If participants did not complete the entire experiment within 2.5 days, their data was automatically rejected by the Gorilla experimental platform. There was a significant difference among the groups in the amount of time passed until completion of the memory test [one-way ANOVA: F(2,109) = 3.61, p = 0.03]. In particular, participants in the Reading group (32.57 ± 10.20 hours) took slightly longer to complete the test than participants in the Listening and Reading group (27.53 ± 6.2 hours; posthoc t-test with Tukey correction was significant with p_tukey_ = 0.026). No significant difference was found between the Listening group (29.38 ±7.45 hours) and the Reading (p_tukey_ = 0.213) or between Listening and Reading groups (p_tukey_ = 0.622).

### Exclusion criteria

To ensure data quality and sufficient trials for analysis, we planned to exclude participants if their learning performance was below 25% for words encountered on Day 1 for M+ trials (as in Ripollés et al., 2014, 2016, 2018). However, no participants were excluded on the basis of scoring less than 25% on Day 1 for M+ trials.

For the Day 2 memory test, we only analysed correct answers. Answers that had a very low confidence rating at recognition (=1) were treated as a guess and were not included in the analysed trials (as in Bains et al., 2024). Of the 4480 total trials from Day 1, 2259 trials were excluded because the word had not been correctly learned or rejected on Day 1, and a further 165 trials were excluded because confidence on Day 2 was 1. This resulted in 2,056 trials included in the memory analyses. Due to these exclusions, in the memory analyses, we analysed M+ data from 110 participants (Listening, N = 36; Reading, N = 41, Reading and Listening, N = 33) and M– data from 97 participants (Listening, N = 32; Reading, N = 37, Reading and Listening, N = 28).

### Data, materials, and code

All data and code have been made publicly available at the OSF and can be accessed at https://osf.io/e57u3/.

### Analyses

Generalised mixed-effects models were fitted to accuracy, memory, and enjoyment data using the *glmer()* function from the *lme4* package (Bates et al., 2014) within R (R Core Team, 2020). Accuracy and memory were analysed using logistic models, and enjoyment ratings were analysed using linear mixed-effects models. Across models examining modality effects, Reading was used as the reference level to facilitate comparison with previous work. To estimate the best-fitting random structure for each model, the *buildmer()* function from the *buildmer* package [version 2.12] (Voeten, 2023) was used. A maximal structure was fitted to the data before applying a backwards elimination process based on the significance of the change in log likelihood between models. For all models (confirmatory and exploratory), we report regression coefficients (b), standard errors (SE), 95% confidence intervals (95% CI), and t/z-values, where |t| > 1.96 and |z| > 1.96 indicate a statistically significant effect.

#### Learning performance

Learning accuracy on Day 1 was calculated separately for congruent (M+) and incongruent (M–) trials. For descriptive purposes, we report the percentage of words correctly learned at encoding in each congruency and modality condition. Inferential analyses tested whether Congruency, Modality, and their interaction predicted learning accuracy using logistic mixed-effects models. The initial model structure was: Accuracy ~ Congruency*Modality + (1+ Congruency | ID) + (1+ Congruency+Modality+List | Item). The best-fitting random effects structure was determined via the *buildmer*() function within R.

#### Memory performance

Memory was operationalised as the correct recognition of word meanings 24 hours after learning. To ensure that analyses reflected memory for successfully encoded representations, we (i) included only words that had been correctly inferred in the M+ condition (or correctly rejected in the M– condition) on Day 1, and (ii) retained only trials for which Day 2 recognition confidence exceeded 1. For descriptive purposes, we report the percentage of words correctly recognised on Day 2 across congruency and modality. Inferential analyses examined whether memory was predicted by Congruency, Modality, and their interaction using logistic mixed-effects models with the initial structure: Memory ~ Congruency *Modality + (1+ Congruency | ID) + (1+ Congruency +Modality+List | Item). The best-fitting random effects structure was determined via the buildmer() function within R.

#### Planned analyses

##### Testing hypothesis 1: Across modalities, we will observe higher enjoyment ratings when participants successfully extract word meanings

Descriptive statistics are reported in Appendix 3, Table A3.1. To test hypothesis 1, we fitted a linear mixed effects model with enjoyment as the dependent variable. We included fixed effects of learning condition (i.e., congruency; M+/M–), modality (reading, listening, or reading and listening), and accuracy (correct/incorrect), as well as all interactions between these variables. We also included random intercepts for participant and item (accounting for random slopes of the counterbalanced list participants to which were assigned, i.e., list 1 or 2). The model initially fitted to the data was


\[\begin{array}{c}
{\rm Enjoyment}\; {\sim}\; {\rm Congruency} * {\rm Accuracy} * {\rm Modality}  + (1 + {\rm Congruency} + {\rm Accuracy} | {\rm ID})\\
+ (1 + {\rm Congruency} + {\rm Accuracy} + {\rm Modality}+ {\rm List} | {\rm Item})
\end{array}\]

Then, the best-fitting fixed and random effects structure was determined via the buildmer() function within R. We expected to observe a significant interaction effect of sentence congruency and accuracy on enjoyment. Specifically, we expected enjoyment to be higher for M+ correct trials than for incorrect ones, with no difference between correct and incorrect M– trials. We did not expect large differences between modalities (i.e., we did not expect a triple interaction to be significant). We planned to explore the direction of these differences using the emtrends function from the emmeans package. Arousal ratings were analysed following the same methodology as enjoyment ratings.

##### Testing hypothesis 2: Across modalities, enjoyment will be higher for words that are successfully recognised, relative to those that are forgotten

Descriptive statistics are reported in Appendix 3, Table A3.1. To test hypothesis 2, we fit a linear mixed effects model with enjoyment as the dependent variable. We included fixed effects of sentence congruency (M+/M–), modality (reading, listening, or reading and listening), and memory (correct/incorrect), and all interactions between these fixed effects. We also accounted for random intercepts of participant and item, including random slopes of counterbalanced list. We only included trials where word meaning was successfully extracted or incongruency was correctly detected on day 1, and those where confidence on day 2 exceeded 1 (as in Bains et al., 2024). The model initially fitted to the data was:


\[\begin{array}{c}
{\rm Enjoyment} \sim {\rm Congruency} * {\rm Memory} * {\rm Modality} +  (1 + {\rm Congruency} + {\rm Memory} | {\rm ID})\\
+ (1 +  {\rm Congruency} + {\rm Memory} + {\rm Modality} + {\rm List} | {\rm Item})
\end{array}\]

Then, the best-fitting fixed and random effects structure was determined via the buildmer() function within R. Again, we expected to observe an interaction between sentence congruency and memory on enjoyment. In other words, we expected enjoyment to be higher for remembered M+ words (i.e., correct) than for forgotten ones (i.e., incorrect) with no difference between correct and incorrect M– trials. We did not expect large differences between modalities (i.e., we did not expect a triple interaction to be significant). We planned to explore the direction of these differences using the emmeans or emtrends function from the emmeans package. Arousal ratings were analysed following the same methodology as enjoyment ratings.

## Results

### Overall learning performance

**Figure 2:**
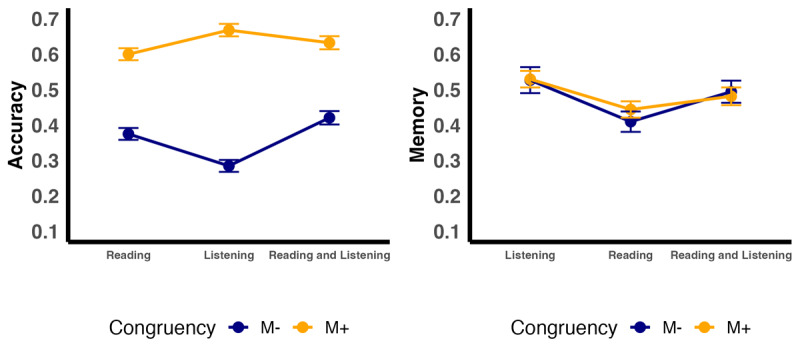
Accuracy and memory by congruency and modality. The figure displays the means of accuracy (left panel) and memory (right panel) across different modalities, separated by Congruency (M– vs. M+). Error bars represent ±1 standard error (SE).

Overall learning performance is shown in Figure 2. For congruent (M+) sentence pairs, participants successfully extracted meaning on 63.2% (SD = 15.5%) of the time (listening, 66.8%, SD = 15.0%; reading, 60%, SD = 16.6%; listening + reading, 63.2%, SD = 14.2%). For the incongruent M– pairs, participants correctly rejected 36.0% (SD = 24.77%) of words (listening, 28.5%, SD = 21.3%; reading, 37.5%, SD = 23.4%; listening + reading, 42.1%, SD = 28.21%). Descriptive patterns are shown in Figure 2.

The model fitted to accuracy data (glmer(Accuracy ~ Congruency + Modality + Congruency:Modality + (1 + Congruency | Participant) + (1 | Item) + (0 + Congruency | Item)) indicated that congruency was a significant predictor of accuracy (see Table 1). Participants were more likely to correctly infer words in the congruent M+ condition, compared to the incongruent M– condition. In addition, the magnitude of the congruency difference differed by Modality, driven by a stronger congruency effect in the Listening condition relative to Reading.

**Table 1:** Generalized Linear Mixed-Effects Results for Accuracy. Z-values in bold indicate that the effect is significant. The final model fitted to the accuracy data was Accuracy ~ Congruency + Modality + Congruency:Modality + (1 + Congruency | Participant) + (1 | Item) + (0 + Congruency | Item).


MEASURE	FIXED EFFECT	b	SE	95% CI	z-value	p-value

Memory	(intercept)	–0.06	0.12	–.29,.17	–0.52	0.602

	Congruency	–0.50	0.07	–.64, –.36	**–7.00**	**<.001**

	Modality: Listening	–0.06	0.14	–.34, .21	–0.45	0.652

	Modality: Listening + Reading	0.20	0.14	–.08,.47	1.40	0.161

	Congruency * Modality (Listening)	–0.40	0.08	–.56, –.24	**–4.96**	**<.0001**

	Congruency * Modality (Listening + Reading)	0.01	0.08	–.15, .17	0.11	0.912

**RANDOM EFFECTS**				**VARIANCE**	**SD**	**CORRELATION**

Participant	(intercept)			0.255	0.505	

Word	(intercept)			0.112	0.334	

	Type: M–			0.083	0.288	

	Type: M+			0.241	0.491	–.15


### Overall memory performance

Participants remembered 49.6% (SD = 20.10%) of words they had correctly learned in the congruent M+ condition on Day 1 (Listening, 53.4%, SD = 21.6%; Reading, 46.40%, SD = 18.40%; Listening + Reading, 49.40%, SD = 20.4%), also see Figure 2. Recognition memory for M+ words on Day 2 was significantly above chance level (one sample t-tests using a test value of 33.33: p < 0.001). In the incongruent M– condition, participants correctly rejected 43.7% (SD = 26.6%) of words they had accurately identified as incongruent on Day 1 [Listening, 44.7%, SD = 32.4%; Reading, 41.1%, SD = 23.5%; Listening + Reading, 45.9%, SD = 23.7%]. Recognition memory for M– words on Day 2 was significantly above chance level for all modalities (p <= .05).

The final model fitted to memory data (glmer(Memory ~ Congruency + Modality + Congruency:Modality + (1 + Congruency | Participant) + (1 + Congruency | Item)) indicated that modality was a significant predictor of memory (see Table 2). Specifically, memory was higher in the listening modality relative to reading. The main effect of Congruency was not significant, nor were the Modality × Congruency interactions.

**Table 2:** Generalized Linear Mixed-Effects Results for Memory. The final model fitted to the memory data was Memory ~ Congruency + Modality + Congruency:Modality + (1 + Congruency | Participant) + (1 + Congruency | Item).


MEASURE	FIXED EFFECT	b	SE	95% CI	z-value	p-value

Accuracy	(Intercept)	–0.11	0.07	–.25, .02	–1.64	0.102

	Congruency (incongruent)	–0.05	0.08	–.21, .11	–0.60	0.547

	Modality: Listening	–0.20	0.09	–.38, –.02	**–2.22**	**0.026**

	Modality: Listening + Reading	0.16	0.10	–.31, .35	1.64	0.101

	Congruency * Modality (Listening)	–0.04	0.09	–.22, .15	–0.41	0.683

	Congruency * Modality (Listening + Reading)	–0.03	0.10	–.23,.16	–0.34	0.732

**RANDOM EFFECTS**				**VARIANCE**	**SD**	**CORRELATION**

Participant	(intercept)			0.160	0.400	

	Congruency			0.199	0.446	0.18

Word	(intercept)			0.005	0.068	

	List 1			0.087	0.294	0.02


#### Planned analyses

##### Testing hypothesis 1: Enjoyment ratings will be higher when word meanings are successfully extracted across modalities

**Figure 3:**
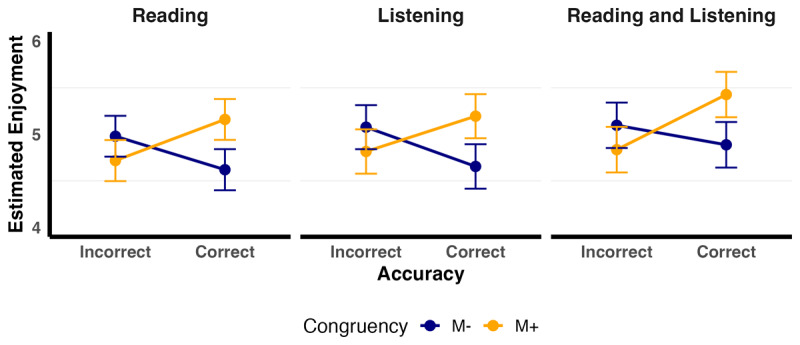
Estimated marginal means of enjoyment by Accuracy, Congruency and Modality. The figure displays the estimated marginal means (EMMs) of enjoyment as a function of Accuracy (“Incorrect” vs. “Correct”) and Congruency (M– vs. M+), with separate panels for each Modality. Error bars show ±1 standard error (SE).

The final model fitted to Enjoyment data (glmer(Enjoyment ~ Congruency + Accuracy + Modality + Congruency:Accuracy + Accuracy:Modality + (1 | Participant) + (1 | Item)) indicated that congruency significantly predicted enjoyment (see Table 3 and Figure 3). Critically, our predicted two-way interaction between congruency and accuracy, was significant. Regardless of modality, words in the M+ correct condition (M_estimated_ = 5.26, SE_estimated_ = 0.14) were enjoyed more than: (1) words in the M+ incorrect condition (M_estimated_ = 4.79, SE_estimated_ = 0.14; z = 9.97, p < .0001); (2) words in the M– correct condition (M_estimated_ = 4.72, SE_estimated_ = 0.14); z = 11.26, p < .0001); and (3) the M– incorrect condition (M_estimated_ = 5.05, SE_estimated_ = 0.14; z = 5.26, p < .0001). The effect of modality was not significant.

**Table 3:** Generalized Linear Mixed-Effects Results for Enjoyment using Day 1 Accuracy. The final model fitted to the accuracy data was Enjoyment ~ Congruency + Accuracy + Modality + Congruency:Accuracy + Accuracy:Modality + (1 | Participant) + (1 | Item).


MEASURE	FIXED EFFECT	b	SE	95% CI	z-value	p-value

Enjoyment	(Intercept)	4.87	0.22	4.44, 5.30	**22.40**	**<.0001**

	Congruency	–0.07	0.02	–0.10, –0.04	**–4.27**	**<.0001**

	Accuracy	–0.02	0.03	–0.07, 0.03	–0.79	0.432

	Modality (Reading and Listening)	0.19	0.32	–0.45, 0.83	0.59	0.553

	Modality (Listening)	0.07	0.32	–0.57, 0.70	0.21	0.836

	Congruency * Accuracy	0.20	0.02	0.17, 0.23	**11.70**	**<.0001**

	Accuracy*Modality (RL)	–0.08	0.04	–0.15, 0.00	–1.90	0.057

	Accuracy*Modality (L)	0.03	0.04	–0.04, 0.11	0.81	0.416

**RANDOM EFFECTS**				**VARIANCE**	**SD**	

Participant	(intercept)			1.94	1.39	

Word	(intercept)			0.01	0.09	


##### Testing hypothesis 2: Memory for word meanings will be predicted by enjoyment across modalities

**Figure 4:**
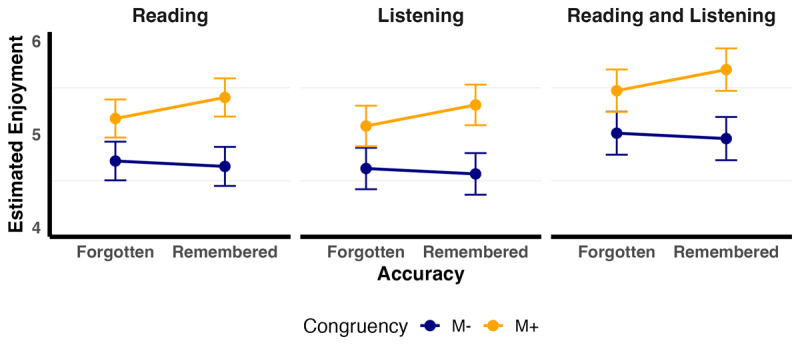
Estimated marginal means of enjoyment by Memory, Congruency and Modality. The figure displays the estimated marginal means (EMMs) of enjoyment as a function of Memory (“Forgotten” vs. “Remembered” 24 hours later) and Congruency (M– vs. M+), with separate panels for each Modality (however, note there is no difference by modality). Error bars show ±1 standard error (SE).

The final model fitted to Enjoyment data (glmer(Enjoyment ~ Congruency + Memory + Modality + Congruency:Memory + (1 | Participant) + (1 | Item)) indicated that congruency influenced enjoyment (see Table 4, Figure 4). We also observed a significant interaction between congruency and memory. M+ words that were correctly remembered (M = 5.46, SE = 0.13) were enjoyed more than: (1) M+ words that were forgotten (M = 5.25, SE = 0.13; z = 3.58, p = .002); words in the M– correct condition that were correctly remembered as incongruent on Day 2 (M = 4.72, SE = 0.14; z = 9.52, p < .0001); and (3), M– incorrect words on day 2 (M = 4.77, SE = 0.14; z = 9.21, p < 0.0001). We did not observe any other significant main effects or interactions.

**Table 4:** Generalized Linear Mixed-Effects Results for Enjoyment using Day 2 Memory. The final model fitted to the memory data was Enjoyment ~ Congruency + Memory + Modality + Congruency:Memory + (1 | Participant) + (1 | Item).


MEASURE	FIXED EFFECT	b	SE	95% CI	z-value	p-value

Enjoyment	(Intercept)	4.98	0.20	4.59, 5.38	**25.10**	**<.0001**

	Congruency	–0.30	0.03	–.35, –.25	**–11.00**	**<.0001**

	Memory	–0.04	0.03	–.09, .01	–1.60	0.110

	Modality (Reading and Listening)	–0.08	0.29	–.66, .49	–0.28	0.782

	Modality (Listening)	0.30	0.30	–.29, .89	1.01	0.316

	Congruency * Accuracy	0.07	0.03	.02, .12	**2.70**	**0.007**

**RANDOM EFFECTS**				**VARIANCE**	**SD**	

Participant	(intercept)			1.54	1.24	

Word	(intercept)			0.01	0.10	


### Control analyses

A linear mixed-effects model was fit to examine the effects of congruency (M+/M–), Accuracy (correct/incorrect), and modality on tiredness ratings (i.e., arousal). This analysis was performed to assess whether the reported effects are specific to reward. We observed a significant interaction between congruency and arousal, suggesting a small increase in arousal for successfully inferred congruent words (fixed effects are shown in Appendix 5; also see Figure A5.1). We also fit a linear mixed-effects model to examine the effects of congruency (M+/M–), Memory (correct/incorrect 24 hours later), and modality on tiredness ratings. We observed no significant main effects of congruency and accuracy. There was a main effect of condition, and the interaction between accuracy and modality was significant (fixed effects are shown in Appendix 5; also see Figure A5.2).

### Exploratory analyses

**Figure 5:**
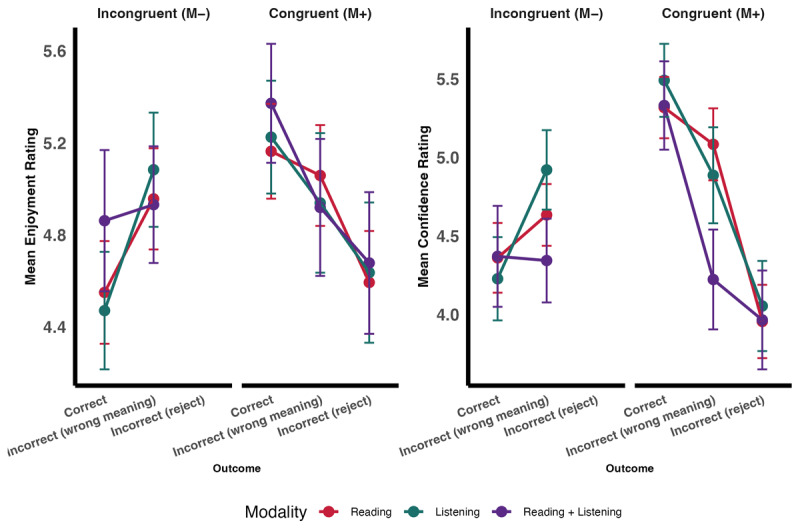
Enjoyment and confidence by congruency, outcome, and modality. The figure displays the means of enjoyment (left panel) and confidence (right panel) separated by Congruency (M– vs. M+) and outcome (Correct, Incorrect meaning inferred, and incorrectly rejected). The different modalities are shown in different colouts (Red: Reading, Green: Listening and Purple: Reading and Listening). Error bars represent ±1 standard error (SE).

**Figure 6:**
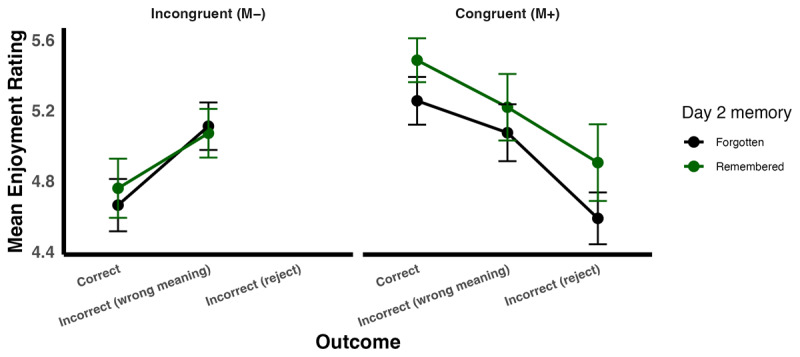
Enjoyment and confidence by congruency, outcome and memory. The figure displays the means of enjoyment separated by Congruency (M– vs. M+) and outcome (Correct, Incorrect meaning inferred, and incorrectly rejected). The different memory conditions are shown in different colours (Black: Forgotten, Green: Remembered). Error bars represent ±1 standard error (SE).

From a participant’s subjective perspective, assigning any meaning to a novel word may feel like learning, regardless of whether that meaning is objectively correct. Such subjective experiences of learning could plausibly influence enjoyment ratings. To examine this possibility, we conducted exploratory analyses that distinguished between different types of incorrect responses. Specifically, M+ trials were divided into those in which participants correctly inferred the meaning, inferred an incorrect meaning, or incorrectly rejected the word. M– trials were divided into those in which participants correctly rejected the word and those in which they incorrectly inferred a meaning. These analyses revealed that inferring a meaning, even when incorrect, was generally associated with higher enjoyment than rejecting a word. Crucially, correctly inferring the meaning of M+ words was associated with the highest enjoyment overall (Figure 5). This graded pattern suggests that enjoyment increases with perceived information gain and is strongest when that inference is also supported by the contextual evidence. Indeed, this pattern was mirrored in confidence ratings (Figure 5), where participants were most confident about words they successfully inferred in the M+ condition. Finally, there was also an indication that this pattern persisted when examining which words were remembered vs. forgotten, with the highest enjoyment observed for words that were correctly remembered (Figure 6).

## Discussion

In line with our hypotheses, we found that intrinsic reward, assessed through behavioural ratings of enjoyment, was related to successful word learning across written and oral contexts, that is, in all three modality conditions: reading, listening, and the combination of reading and listening. This is the first demonstration of the link between intrinsic reward and word learning from context in an oral context, paving the way for new research avenues, especially in the developmental domain where language acquisition mostly occurs in oral contexts (Flack et al., 2018; Horst et al., 2011). Crucially, across the three modalities, we find that M+ words that are remembered the next day are associated with greater enjoyment. This indicates that the link between intrinsic reward and memory is robust, exists across sensory modalities, and deserves further attention.

### The relationship between reward and successful extraction of word meaning

Our finding that intrinsic reward is linked to the successful extraction of word meaning and its mapping to a new word-form (i.e., word learning: the memory test scores show that participants were not only able to extract the meaning of a new word, but were also able to link it to a new word form and to recognise this mapping after 24h) is consistent with our previous work on word learning from written context (Angwin et al., 2019; Bains et al., 2024; Ripollés et al., 2014, 2016, 2018; Vavra et al., 2023). Here, we now demonstrate this relationship in the auditory and auditory-visual domains (i.e., in oral contexts). Our results indicate that the strength of the relationship between increased reward and successful word learning is similar across modalities. This strongly suggests that the link between reward (measured through behavioral ratings of enjoyment) and word learning exists independent of modality. Critically, the extension of the previously identified relationship between reward and word learning to the auditory domain is expected but not guaranteed. For instance, some individuals experience specific music anhedonia, a condition where they derive reward from typically rewarding stimuli (e.g., food) but not from music, an auditory reinforcer (Mas-Herrero et al., 2013, 2014). The ability to learn words from oral contexts aligns with predictions that this relationship plays a crucial role in language development throughout early stages of life (Bains et al., 2024), and through evolution (Syal & Finlay, 2011). Establishing that this effect is modality-independent means we can examine links between reward and language learning in populations that do not read, such as younger children or adults who are illiterate, or those who have difficulty reading and benefit from orthographic facilitation.

It is rather unlikely that the experience of intrinsic reward for learning is confined to word learning. Rather, we argue that word learning benefits from a reward-seeking process that occurs across domains to aid learners (Becker & Cabeza, 2025). For example, Kizilirmac and colleagues (2016) have demonstrated that insight into a learning problem can benefit long-term memory. They investigated this through trying to identify objects in ambiguous black and white photos. When participants had an “aha” moment, they provided higher affective ratings, indicating a more rewarding experience. In a similar vein, we would predict that understanding of noise-vocoded sentences that were previously unintelligible (e.g., Alderson-Day et al., 2017) would lead to a similarly rewarding experience. This contextual word learning paradigm simply offers a relatively naturalistic means to investigate this relationship for language (although we note that the explicit nature of our task, where the word learning component is made explicit and participants provide ratings, does somewhat attenuate the naturalistic nature of contextual word learning).

In line with this reward-seeking hypothesis, we argue that the experience of intrinsic reward is not simply due to problem solving, but information gain – or more precisely, learning the mapping between a new word and its meaning. The relationship between reward and word learning was quite specific, as it was only observed for the M+ trials, where correct extraction of word meaning was possible. For the M– trials, where it was possible to answer correctly without extracting meaning, we did not observe a positive relationship between intrinsic reward and accuracy. In other words, increased accuracy (i.e., successfully detecting the incongruent meaning evoked by the M-sentences) was not related to an increased sense of reward, despite the successful completion of the task. This suggests that other factors such as difficulty, novelty, or the mere successful completion of a task are not sufficient drivers of this relationship: in our paradigm, increased reward was specific to correctly extracting a meaning during the congruent condition (i.e., learning a new item). This suggests that reward, in our word learning paradigm, is closely tied to information gain. Indeed, this is shored up by our exploratory analyses. The graded pattern we observe based on perceived accuracy suggests that enjoyment increases with perceived information gain. Further, supporting this idea, successful learning in the congruent condition was also accompanied by the highest confidence ratings. Indeed, in the absence of any explicit feedback, participants use confidence signals to generate their own feedback (Guggenmos et al., 2016).

### The relationship between reward and memory for words

Intrinsic reward, measured through ratings of enjoyment, was linked to memory for words across all three modalities. Our findings offer a convincing replication of this relationship in written contexts (Ripollés et al., 2016, 2018) and extend this work to suggest that the relationship between reward and memory for new words is agnostic to the modality in which words are encountered. This finding aligns with other studies exploring intrinsic reward-related signals, such as curiosity and music reward, which similarly show that stronger reward-related signals are linked to improved memory (Curzel et al., 2024; Ferreri et al., 2021; Garvin & Krishnan, 2022; Gruber et al., 2014; Kang et al., 2009; Marvin & Shohamy, 2016).

This association is likely to be driven by known links between reward and memory circuits, wherein anticipation of large explicit rewards strengthens the functional connectivity between striatal and midbrain dopaminergic reward regions and hippocampal memory systems even before the material to be learned is presented (Adcock et al., 2006). Research in the domain of music reward further supports the idea that material presented in the auditory domain (such as here in the Listening and Listening + Reading condition) can engage reward mechanisms. Studies show that pleasurable music can modulate functional connectivity between auditory regions and reward-related, dopaminergic regions (Martínez-Molina et al., 2016; Mori & Zatorre, 2024; Salimpoor et al., 2013). Ripollés and colleagues (2016) have shown that successful extraction of word meaning also activates a dopaminergic circuit which fuels memory for word learning, suggesting that intrinsic reward states activate the same circuits. In non-linguistic domains, the link between affective and memory processes that occurs when participants gain insight into a solution is recognized as the insight memory advantage (Danek & Wiley, 2020). This is thought to result from the positive affective response people receive at the moment of epiphany (Danek & Wiley, 2020; Kizilirmak et al., 2016). This speaks to the possibility that intrinsic reward could be tapped as a means of boosting learning. One way to do this might be to restructure situations to explicitly facilitate generation of hypotheses about the meaning of new words, rather than simply presenting meanings to participants.

It is important to highlight that our task difficulty was carefully controlled: we aimed to reach an overall accuracy level on day 1 of around 60% for M+ trials. We therefore ensured that our block structure was complex, with novel words being presented non-sequentially as it happens in real life contextual word learning (at least four sentences apart; see Figure 1). This is because some level of difficulty appears necessary to drive the activation dopaminergic systems associated with learning, perhaps as the affective response to problem solving does not appear without such difficulty (Vavra et al., 2023). This would suggest that it is not necessary for educational material to be perceived as easy, as some difficulty might be desirable.

Finally, we observed a small increase in arousal during successful learning of congruent words, which is consistent with the possibility of noradrenergic engagement during learning (Becker & Cabeza, 2025). However, successfully remembered words were not associated with higher arousal at encoding. This dissociation suggests that the relationship between reward-related processes and later memory may be more specific than a general increase in arousal.

### The influence of modality on memory for words

Most previous research suggests that providing the written form of a word facilitates word learning, for example when watching movies with subtitles (Linebarger et al., 2010), for second language learners (Vidal, 2011; Brown et al., 2008), and in children (Clark & Reuterskiöld, 2021; Ricketts et al., 2009). However, our results show that people in the reading + listening modality did not learn more than those in the reading or listening modalities. This may reflect differences in the task – our participants were also neurotypical adults and word forms to be learned conformed to English phonotactics. Further, we focused on the mapping between a new word and existing meaning. Presentation of a written word may be particularly helpful when learning phonology, for example, in tasks that require participants to remember or spell the new word form.

### Limitations and future directions

In our study, we asked people to rate their enjoyment as a proxy for intrinsic reward. Previous studies have revealed that these ratings converge with other measures of the dopaminergic reward system, such as electrodermal activity, pharmacological interventions, or brain activity (Ripolles et al., 2014, 2016, 2018). In future work, it would be useful to assess whether such measures converge with our behavioural findings. MRI studies could also be helpful in establishing whether the representations that are being accessed are truly independent of modality (Evans et al., 2019).

Furthermore, the memory analysis was based on 60% of the data for M+ trials and 36% for M– trials, creating an imbalance that may limit the reliability of direct comparisons between conditions. This disparity primarily reflects lower learning accuracy in the M– condition, which offers a surprising contrast with previous findings. Prior studies have generally reported similar learning rates across M+ and M– trials. For example, when comparable materials were used with children aged 10–18 years (Bains et al., 2024), performance was equivalent across congruency conditions (M+: 74.5%; M–: 73.8%). Likewise, using English materials closely matched to ours, Angwin et al. (2019) observed slightly higher learning accuracy for M– trials (98%) than M+ trials (66%) in adults. Notably, in both these studies, sentences were presented sequentially. Taken together, these findings suggest that the reduced M– accuracy observed here is not driven by the sentence materials themselves, and instead reflects the non-sequential presentation format, which increases the likelihood of failing to correctly reject M– trials.

There was also variability in the time elapsed between the learning phase and the memory test across groups. Specifically, participants in the Reading group took slightly longer to complete the test compared to those in the Listening and Reading group. To address this issue, we conducted an exploratory analysis excluding those with long delay times (Appendix 3). Reassuringly, we were able to replicate the same pattern of results.

Another limitation of this study is the restricted choice of words to be learned, which were limited to concrete nouns (e.g., “graveyard”). While this approach is methodologically sound, it does not reflect the complexity of natural language, where many words are abstract or polysemous (i.e., having one-to-many mappings). Future studies should explore whether the effects observed in this study extend to the learning of words other than concrete nouns. Finally, this was a between-subject design, which means there are potential differences by group. Even if there were no differences between groups in terms of demographic factors and sensitivity to reward, these results should be replicated using a within-subject design.

Our results suggest that eliciting intrinsic reward could help to drive novel word learning, which might be relevant for boosting the learning of words in a foreign language. Here, we have focused on neurotypical English-speaking adults. The pseudowords we used followed English phonotactics and were embedded in English sentences. It is therefore important to assess whether these findings would hold for words with different phonotactic structure, or when people were less fluent with the language that the words were embedded in. It is also important to establish whether these findings might generalize to populations with poorer word learning, such as children with developmental language disorder or dyslexia (Nation, 2014). If so, this could indicate a powerful mechanism to boost learning.

### Summary and conclusions

Our results demonstrate that successful extraction of novel word meanings is perceived as intrinsically rewarding, regardless of the modality words are encountered in. Additionally, this experience of intrinsic reward fuels memory for words. This indicates a robust reward-language link which is independent of sensory modality and that has the potential to be tapped to boost word learning in educational contexts.

## Additional Files

The additional files for this article can be found as follows:

10.5334/joc.499.s1 Appendix 1. Post-hoc sensitivity analyses.

10.5334/joc.499.s2 Appendix 2. Cloze Probability Norming Study.

10.5334/joc.499.s3 Appendix 3. Descriptive Statistics for ratings obtained on Day 1.

10.5334/joc.499.s4 Appendix 4. Memory analyses controlling for delay time.

10.5334/joc.499.s5 Appendix 5. The effect on arousal ratings.

## Data Availability

Data and code for this experiment is openly available on the Open Science Framework at https://osf.io/e57u3/.

## References

[1] Adcock, R. A., Thangavel, A., Whitfield-Gabrieli, S., Knutson, B., & Gabrieli, J. D. E. (2006). Reward-Motivated Learning: Mesolimbic Activation Precedes Memory Formation. Neuron, 50(3), 507–517. 10.1016/j.neuron.2006.03.036 16675403

[2] Akhtar, N. (2004). Contexts of Early Word Learning. In D. G. Hall & S. Waxman (Eds.), Weaving a lexicon (pp. 485–507). MIT Press. https://psycnet.apa.org/record/2004-12698-015

[3] Alderson-Day, B., Lima, C. F., Evans, S., Krishnan, S., Shanmugalingam, P., Fernyhough, C., & Scott, S. K. (2017). Distinct processing of ambiguous speech in people with non-clinical auditory verbal hallucinations. Brain, 140(9), 2475–2489. 10.1093/brain/awx206 29050393

[4] Angwin, A. J., Wilson, W. J., Ripollés, P., Rodriguez-Fornells, A., Arnott, W. L., Barry, R. J., Cheng, B. B. Y., Garden, K., & Copland, D. A. (2019). White noise facilitates new-word learning from context. Brain and Language, 199, 104699. 10.1016/j.bandl.2019.104699 31569040

[5] Anwyl-Irvine, A. L., Massonnié, J., Flitton, A., Kirkham, N., & Evershed, J. K. (2020). Gorilla in our midst: An online behavioral experiment builder. Behavior Research Methods, 52(1), 388–407. 10.3758/s13428-019-01237-x 31016684 PMC7005094

[6] Bains, A., Barber, A., Nell, T., Ripollés, P., & Krishnan, S. (2024). The role of intrinsic reward in adolescent word learning. Developmental Science, 27, e13513. 10.1111/desc.13513 38685611

[7] Bates, D., Mächler, M., Bolker, B., & Walker, S. (2014). Fitting Linear Mixed-Effects Models using lme4. arXiv:1406.5823 [Stat]. https://arxiv.org/abs/1406.5823

[8] Becker, M., & Cabeza, R. (2025). The neural basis of the insight memory advantage. Trends in Cognitive Sciences. 10.1016/j.tics.2025.01.001 39863514

[9] Bisson, M.-J., Kukona, A., & Lengeris, A. (2021). An ear and eye for language: Mechanisms underlying second language word learning. Bilingualism: Language and Cognition, 24(3), 549–568. 10.1017/S1366728920000723

[10] Brown, R., Waring, R., & Donkaewbua, S. (2008). Incidental Vocabulary Acquisition from Reading, Reading-While-Listening, and Listening to Stories. Reading in a Foreign Language, 20. 10.64152/10125/66816

[11] Campbell, J. I. D., & Thompson, V. A. (2012). MorePower 6.0 for ANOVA with relational confidence intervals and Bayesian analysis. Behavior Research Methods, 44(4), 1255–1265. 10.3758/s13428-012-0186-0 22437511

[12] Clark, G. T., & Reuterskiöld, C. (2021). Orthographic Support for Word Learning in Clinical Populations: A Systematic Review. Language, Speech, and Hearing Services in Schools, 52(3), 937–948. 10.1044/2021_LSHSS-20-00123 34029128

[13] Curzel, F., Osiurak, F., Trân, E., Tillmann, B., Ripollés, P., & Ferreri, L. (2024). Enhancing musical pleasure through shared musical experience. iScience, 27(6). 10.1016/j.isci.2024.109964 PMC11145343 38832017

[14] Danek, A. H., & Wiley, J. (2020). What causes the insight memory advantage? Cognition, 205, 104411. 10.1016/j.cognition.2020.104411 32762872

[15] Dickerson, K. C., & Adcock, R. A. (2018). Motivation and Memory. In J. T. Wixted (Ed.), Stevens’ Handbook of Experimental Psychology and Cognitive Neuroscience (1st ed., pp. 1–36). Wiley. 10.1002/9781119170174.epcn107

[16] Elley, W. B. (1989). Vocabulary Acquisition from Listening to Stories. Reading Research Quarterly, 24(2), 174. 10.2307/747863

[17] Evans, S., Price, C. J., Diedrichsen, J., Gutierrez-Sigut, E., & MacSweeney, M. (2019). Sign and speech share partially overlapping conceptual representations. Current Biology, 29(21), 3739–3747. 10.1016/j.cub.2019.08.075 31668623 PMC6839399

[18] Ferreri, L., Mas-Herrero, E., Cardona, G., Zatorre, R. J., Antonijoan, R. M., Valle, M., Riba, J., Ripollés, P., & Rodriguez-Fornells, A. (2021). Dopamine modulations of reward-driven music memory consolidation. Annals of the New York Academy of Sciences, 1502(1), 85–98. 10.1111/nyas.14656 34247392

[19] Flack, Z. M., Field, A. P., & Horst, J. S. (2018). The effects of shared storybook reading on word learning: A meta-analysis. Developmental Psychology, 54(7), 1334–1346. 10.1037/dev0000512 29595311

[20] Garvin, B., & Krishnan, S. (2022). Curiosity-driven learning in adults with and without dyslexia. Quarterly Journal of Experimental Psychology, 75(1), 156–168. 10.1177/17470218211037474 PMC8600593 34293988

[21] Gruber, M. J., Gelman, B. D., & Ranganath, C. (2014). States of Curiosity Modulate Hippocampus-Dependent Learning via the Dopaminergic Circuit. Neuron, 84(2), 486–496. 10.1016/j.neuron.2014.08.060 25284006 PMC4252494

[22] Guggenmos, M., Wilbertz, G., Hebart, M. N., & Sterzer, P. (2016). Mesolimbic confidence signals guide perceptual learning in the absence of external feedback. Elife, 5, e13388. 10.7554/eLife.13388.019 27021283 PMC4821804

[23] Henderson, L., Devine, K., Weighall, A., & Gaskell, G. (2015). When the daffodat flew to the intergalactic zoo: Off-line consolidation is critical for word learning from stories. Developmental Psychology, 51(3), 406–417. 10.1037/a0038786 25642602

[24] Horst, J. S., Parsons, K. L., & Bryan, N. M. (2011). Get the Story Straight: Contextual Repetition Promotes Word Learning from Storybooks. Frontiers in Psychology, 2. 10.3389/fpsyg.2011.00017 PMC3111254 21713179

[25] Jamil, T., Ly, A., Morey, R. D., Love, J., Marsman, M., & Wagenmakers, E.-J. (2017). Default “Gunel and Dickey” Bayes factors for contingency tables. Behavior Research Methods, 49(2), 638–652. 10.3758/s13428-016-0739-8 27325166 PMC5405059

[26] Kang, M. J., Hsu, M., Krajbich, I. M., Loewenstein, G., McClure, S. M., Wang, J. T., & Camerer, C. F. (2009). The Wick in the Candle of Learning: Epistemic Curiosity Activates Reward Circuitry and Enhances Memory. Psychological Science, 20(8), 963–973. 10.1111/j.1467-9280.2009.02402.x 19619181

[27] Keuleers, E., & Brysbaert, M. (2010). Wuggy: A multilingual pseudoword generator. Behavior Research Methods, 42(3), 627–633. 10.3758/BRM.42.3.627 20805584

[28] Kizilirmak, J. M., Galvao Gomes da Silva, J., Imamoglu, F., & Richardson-Klavehn, A. (2016). Generation and the subjective feeling of “aha!” are independently related to learning from insight. Psychological Research, 80(6), 1059–1074. 10.1007/s00426-015-0697-2 26280758 PMC5069302

[29] Lee, M. D., & Wagenmakers, E.-J. (2014). Bayesian Cognitive Modeling: A Practical Course. Cambridge University Press. 10.1017/CBO9781139087759

[30] Lenhart, J., Lenhard, W., Vaahtoranta, E., & Suggate, S. (2018). Incidental vocabulary acquisition from listening to stories: A comparison between read-aloud and free storytelling approaches. Educational Psychology, 38(5), 596–616. 10.1080/01443410.2017.1363377

[31] Linebarger, D., Piotrowski, J. T., & Greenwood, C. R. (2010). On-screen print: The role of captions as a supplemental literacy tool. Journal of Research in Reading, 33(2), 148–167. 10.1111/j.1467-9817.2009.01407.x

[32] Lisman, J., & Grace, A. A. (2005). The Hippocampal-VTA Loop: Controlling the Entry of Information into Long-Term Memory. Neuron, 46(5), 703–713. 10.1016/j.neuron.2005.05.002 15924857

[33] Lisman, J., Grace, A. A., & Duzel, E. (2011). A neoHebbian framework for episodic memory; role of dopamine-dependent late LTP. Trends in Neurosciences, 34(10), 536–547. 10.1016/j.tins.2011.07.006 21851992 PMC3183413

[34] Love, J., Selker, R., Marsman, M., Jamil, T., Dropmann, D., Verhagen, J., … & Wagenmakers, E. J. (2019). JASP: Graphical statistical software for common statistical designs. Journal of Statistical Software, 88, 1–17. 10.18637/jss.v088.i02

[35] Martínez-Molina, N., Mas-Herrero, E., Rodríguez-Fornells, A., Zatorre, R. J., & Marco-Pallarés, J. (2016). Neural correlates of specific musical anhedonia. Proceedings of the National Academy of Sciences, 113(46), E7337–E7345. 10.1073/pnas.1611211113 PMC5135354 27799544

[36] Marvin, C. B., & Shohamy, D. (2016). Curiosity and reward: Valence predicts choice and information prediction errors enhance learning. Journal of Experimental Psychology: General, 145(3), 266–272. 10.1037/xge0000140 26783880

[37] Mas-Herrero, E., Marco-Pallares, J., Lorenzo-Seva, U., Zatorre, R. J., & Rodriguez-Fornells, A. (2013). Individual Differences in Music Reward Experiences. Music Perception, 31(2), 118–138. 10.1525/mp.2013.31.2.118

[38] Mas-Herrero, E., Zatorre, R. J., Rodriguez-Fornells, A., & Marco-Pallarés, J. (2014). Dissociation between Musical and Monetary Reward Responses in Specific Musical Anhedonia. Current Biology, 24(6), 699–704. 10.1016/j.cub.2014.01.068 24613311

[39] Mestres-Missé, A., Rodriguez-Fornells, A., & Münte, T. F. (2007). Watching the Brain during Meaning Acquisition. Cerebral Cortex, 17(8), 1858–1866. 10.1093/cercor/bhl094 17056648

[40] Mori, K., & Zatorre, R. (2024). State-dependent connectivity in auditory-reward networks predicts peak pleasure experiences to music. PLOS Biology, 22(8), e3002732. 10.1371/journal.pbio.3002732 39133721 PMC11318860

[41] Murayama, K., & Kitagami, S. (2014). Consolidation power of extrinsic rewards: Reward cues enhance long-term memory for irrelevant past events. Journal of Experimental Psychology: General, 143(1), 15–20. 10.1037/a0031992 23421444

[42] Nagy, W. E., Herman, P. A., & Anderson, R. C. (1985). Learning Words from Context. Reading Research Quarterly, 20(2), 233–253. JSTOR. 10.2307/747758

[43] Nation, K. (2014). Lexical learning and lexical processing in children with developmental language impairments. Philosophical Transactions of the Royal Society of London B: Biological Sciences, 369(1634), 20120387. 10.1098/rstb.2012.0387 24324231 PMC3866417

[44] Perez, M. M., Peters, E., Clarebout, G., & Desmet, P. (2018). Effects of Captioning on Video Comprehension and Incidental Vocabulary Learning. Language Learning.

[45] R Core Team. (2020). R: A language and environment for statistical computing. R Foundation for Statistical Computing. https://www.R-project.org/.

[46] Ricketts, J., Bishop, D. V. M., & Nation, K. (2009). Orthographic facilitation in oral vocabulary acquisition. The Quarterly Journal of Experimental Psychology, 62(10), 1948–1966. 10.1080/17470210802696104 19301209

[47] Ripollés, P., Ferreri, L., Mas-Herrero, E., Alicart, H., Gómez-Andrés, A., Marco-Pallares, J., Antonijoan, R. M., Noesselt, T., Valle, M., Riba, J., & Rodriguez-Fornells, A. (2018). Intrinsically regulated learning is modulated by synaptic dopamine signaling. eLife, 7, e38113. 10.7554/eLife.38113 30160651 PMC6133552

[48] Ripollés, P., Marco-Pallarés, J., Alicart, H., Tempelmann, C., Rodríguez-Fornells, A., & Noesselt, T. (2016). Intrinsic monitoring of learning success facilitates memory encoding via the activation of the SN/VTA-Hippocampal loop. eLife, 5, e17441. 10.7554/eLife.17441 27644419 PMC5030080

[49] Ripollés, P., Marco-Pallarés, J., Hielscher, U., Mestres-Missé, A., Tempelmann, C., Heinze, H.-J., Rodríguez-Fornells, A., & Noesselt, T. (2014). The Role of Reward in Word Learning and Its Implications for Language Acquisition. Current Biology, 24(21), 2606–2611. 10.1016/j.cub.2014.09.044 25447993

[50] Rohlfing, K. J., Ceurremans, J., & Horst, J. S. (2018). Benefits of Repeated Book Readings in Children With SLI. Communication Disorders Quarterly, 39(2), 367–370. 10.1177/1525740117692480

[51] Salimpoor, V. N., van den Bosch, I., Kovacevic, N., McIntosh, A. R., Dagher, A., & Zatorre, R. J. (2013). Interactions Between the Nucleus Accumbens and Auditory Cortices Predict Music Reward Value. Science, 340(6129), 216–219. 10.1126/science.1231059 23580531

[52] Shohamy, D., & Adcock, R. A. (2010). Dopamine and adaptive memory. Trends in Cognitive Sciences, 14(10), 464–472. 10.1016/j.tics.2010.08.002 20829095

[53] Swanborn, M. S. L., & De Glopper, K. (1999). Incidental Word Learning While Reading: A Meta-Analysis. Review of Educational Research, 69(3), 261–285. 10.3102/00346543069003261

[54] Syal, S., & Finlay, B. L. (2011). Thinking outside the cortex: Social motivation in the evolution and development of language. Developmental Science, 14(2), 417–430. 10.1111/j.1467-7687.2010.00997.x 22213910

[55] Valentini, A., Ricketts, J., Pye, R. E., & Houston-Price, C. (2018). Listening while reading promotes word learning from stories. Journal of Experimental Child Psychology, 167, 10–31. 10.1016/j.jecp.2017.09.022 29154028

[56] Vavra, P., Sokolovič, L., Porcu, E., Ripollés, P., Rodriguez-Fornells, A., & Noesselt, T. (2023). Entering into a self-regulated learning mode prevents detrimental effects of feedback removal on memory. Npj Science of Learning, 8(1), 2. 10.1038/s41539-022-00150-x 36609382 PMC9823107

[57] Verga, L., & Kotz, S. A. (2013). How relevant is social interaction in second language learning? Frontiers in Human Neuroscience, 7. 10.3389/fnhum.2013.00550 PMC3759854 24027521

[58] Vervoort, L., Vandeweghe, L., Vandewalle, J., Van Durme, K., Vandevivere, E., Wante, L., … & Braet, C. (2015). Measuring Punishment and Reward Sensitivity in children and adolescents with a parent-report version of the Bis/Bas-scales. Personality and Individual Differences, 87, 272–277. 10.1016/j.paid.2015.08.024

[59] Vidal, K. (2011). A Comparison of the Effects of Reading and Listening on Incidental Vocabulary Acquisition. Language Learning, 61(1), 219–258. 10.1111/j.1467-9922.2010.00593.x

[60] Wagenmakers, E. J., Marsman, M., Jamil, T., Ly, A., Verhagen, J., Love, J., … & Morey, R. D. (2018). Bayesian inference for psychology. Part I: Theoretical advantages and practical ramifications. Psychonomic bulletin & review, 25(1), 35–57. 10.3758/s13423-017-1343-3 28779455 PMC5862936

