# Supplementary material for: Intrinsic Reward Modulates Word Learning in Both Oral and Written Contexts
Source: J Cogn. 2026 Apr 30;9(1):28. doi: 10.5334/joc.499 (PMC13131340; doi:10.5334/joc.499)
Supplement: Appendix 1. — Post-hoc sensitivity analyses. [file joc-9-1-499-s1.pdf]

Appendix 1. Post-hoc sensitivity analyses

Sensitivity analyses for the effect of Congruency and Accuracy on Enjoyment

We conducted simulation-based sensitivity analyses to estimate the sensitivity of our design to detect effects of approximately $t > 2$, following recommendations by Kumle et al. (2021). Power was estimated using the *mixedpower* package in R. This approach generates simulated datasets based on the fitted mixed-effects model, refits the model to each simulated dataset, and evaluates whether the target effect reaches statistical significance in each iteration.

We simulated datasets across a range of sample sizes to explore how power scales with the number of participants. For each configuration, the model with data from the reading sample alone was used in the data-generating process, ensuring that the simulated data reflected the observed variance structure and random-effects composition. The proportion of simulations yielding $p < .05$ provided an empirical estimate of statistical power.

We used data from the reading task to fit the initial model, given that previous research using the same task has shown comparable variance structures and participant-level variability (Ripolles et al., 2014, 2016, 2018). The fitted model was:

*lmer(Enjoyment ~ Congruency * Accuracy + (1 | ID) + (1 | Item))*

The same fixed effects were specified for the power analysis, and parameters estimated from this model were used to generate simulated datasets. We varied the number of simulated participants (20–60) to examine how power changes with sample size.

Table A1.1

Effect	***N = 20***	***N = 30***	***N = 40***	***N = 50***
Congruency (MP)	0.69	0.90	0.95	0.98
Accuracy	0.91	0.98	1.00	1.00
Congruency x Accuracy	1.00	1.00	1.00	1.00

Across simulated sample sizes, power was >90% for all fixed effects with 30 participants or more. These results suggest that with 30 participants in each group, the study was well powered to detect effects of the magnitude observed in the data.

It is important to note, however, that this analysis was conducted post hoc—that is, after inspecting the observed data. While simulation-based approaches provide useful insight into design sensitivity, they are not intended as retrospective justifications of statistical power. Because the simulations are based on observed effect sizes and estimated variances, they may overestimate true prospective power. We therefore treat these analyses as illustrative rather than confirmatory.

Power analyses for the effects of Congruency, Modality and Accuracy on Enjoyment

Finally, we estimated power for the three-way interaction between congruency, accuracy and modality. A model using the study data was used to derive estimates of the observed variance structure and random-effects composition, as this was previously unknown. However, we adjusted the effect size of the three-way interaction to be beta = 0.3 (as the obtained effect was not significant).

FLPmodel <- lmer(Enjoyment ~ Congruency * Accuracy * Modality + (1 | ID) + (1 | Item))

Our results suggested that with 110 participants, we had >80% power to detect this effect size.

Table A1.2

Effect	***N = 80***	***N = 90***	***N = 100***	***N = 110***
Congruency (MP)	0.84	0.89	0.90	0.93
Accuracy	1.00	1.00	1.00	1.00
Task: Reading	0.06	0.09	0.08	0.09
Task: Reading and Listening	0.07	0.07	0.07	0.08
Congruency × Accuracy	1.00	1.00	1.00	1.00
Congruency × Task: Reading	0.20	0.23	0.22	0.27
Congruency × Task: Reading and Listening	0.17	0.19	0.20	0.23
Accuracy × Task: Reading	0.33	0.36	0.38	0.44
Accuracy × Task: Reading and Listening	0.82	0.85	0.88	0.92
Congruency × Accuracy × Task: Reading	0.69	0.78	0.81	0.84
Congruency × Accuracy × Task: Reading and Listening	0.67	0.72	0.75	0.82
