# Supplementary material for: Intrinsic Reward Modulates Word Learning in Both Oral and Written Contexts
Source: J Cogn. 2026 Apr 30;9(1):28. doi: 10.5334/joc.499 (PMC13131340; doi:10.5334/joc.499)
Supplement: Appendix 2. — Cloze Probability Norming Study. [file joc-9-1-499-s2.pdf]

Appendix 2. Cloze Probability Norming Study

Ethics

One hundred and ninety-two undergraduate psychology students from Royal Holloway, University of London, took part in the study for course credit. Participants gave informed consent prior to taking part. In line with university policy, all interested students were allowed to complete the task to obtain credits. However, for the purposes of our analyses, we excluded data from all participants who reported being neurodivergent or who spoke English as a second or an additional language. We also excluded data from one participant who did not provide their age, and one participant who provided age-inappropriate responses throughout. We retained data from 144 participants (127 females, 17 males) with an average age of 19.79 years (SD=3.18 years).

Materials

Cloze probability was assessed for all 80 sentences used in the experiment. Each sentence was truncated immediately before the target noun (e.g., *A dog has a keen sense of*___), and participants were asked to type the single word they thought best completed the sentence. No time limit or feedback was provided.

Procedure

The task was completed online via Gorilla.sc. After providing consent and demographic information, participants completed the sentence-completion task. Each participant saw each sentence only once, and sentence order was randomised.

Data processing and analysis

For each sentence, cloze probability was calculated as the proportion of participants who produced the most frequent completion. For example, 135 out of 144 participants completed *A dog has a keen sense of* ___ with “smell”, yielding a cloze probability of 93.75% for smell.

Responses were cleaned prior to analysis: all entries were converted to lowercase, punctuation was removed, and blank responses were excluded. Morphological variants and near-misspellings were counted toward the intended word (e.g., nights → night, nght → night). Responses were manually reviewed to ensure accuracy.

There was missing data for 4 sentences, where we had 143 rather than 144 responses. This did not significantly influence results.

Results

Mean cloze probability across all sentences was 59.48% (SD = 25.92%).

We then computed the cloze probability for the first sentence against the target word of the sentence it was paired with. As expected, sentences used as the first sentence had low cloze probabilities (M=20.34%, SD=19.90%), whereas sentences used as second sentences had high cloze probabilities (M=79.26%, SD=14.88%).

A full list of sentences, their most common completions, total number of responses, and cloze probabilities (for the intended target) is provided on OSF (https://osf.io/n6hac/).
