# Supplementary material for: Intrinsic Reward Modulates Word Learning in Both Oral and Written Contexts
Source: J Cogn. 2026 Apr 30;9(1):28. doi: 10.5334/joc.499 (PMC13131340; doi:10.5334/joc.499)
Supplement: Appendix 3. — Descriptive Statistics for ratings obtained on Day 1. [file joc-9-1-499-s3.pdf]

Appendix 3. Descriptive Statistics for ratings obtained on Day 1

Table A3.1. *Participant-level descriptive statistics for enjoyment, confidence, and arousal ratings categorised by congruency (M+/M-) and Day 1 accuracy (Correct/ Incorrect). Values are means, with standard deviations in parentheses and ranges in square brackets.*

			**Enjoyment**	**Confidence**	**Tiredness**
Reading	M-	Incorrect	4.96 (1.43) [1.00–8.00]	4.63 (1.27) [1.37–7.10]	5.65 (1.19) [2.33–8.00]
	M-	Correct	4.55 (1.39) [1.00–7.00]	4.36 (1.39) [1.00–7.00]	5.52 (1.32) [2.21–8.00]
	M+	Incorrect	4.72 (1.36) [1.00–7.11]	4.30 (1.15) [1.45–6.67]	5.60 (1.31) [1.78–8.00]
	M+	Correct	5.16 (1.34) [1.00–7.88]	5.32 (1.27) [1.44–7.45]	5.63 (1.23) [2.00–8.00]
Reading and Listening	M-	Incorrect	4.93 (1.46) [1.00–8.06]	4.34 (1.53) [1.00–7.94]	5.48 (1.52) [2.44–9.00]
	M-	Correct	4.86 (1.68) [1.00–8.43]	4.37 (1.76) [1.00–7.25]	5.71 (1.56) [2.00–9.00]
	M+	Incorrect	4.85 (1.58) [1.00–8.00]	4.17 (1.59) [1.00–8.00]	5.53 (1.73) [2.25–9.00]
	M+	Correct	5.37 (1.51) [1.00–8.08]	5.33 (1.64) [1.00–8.08]	5.64 (1.50) [2.75–9.00]
Listening	M-	Incorrect	5.08 (1.49) [1.62–8.95]	4.92 (1.52) [2.00–9.00]	5.00 (1.59) [1.56–9.00]
	M-	Correct	4.47 (1.45) [1.75–9.00]	4.23 (1.50) [2.00–9.00]	4.81 (1.53) [1.75–9.00]
	M+	Incorrect	4.70 (1.64) [1.00–9.00]	4.45 (1.61) [1.88–9.00]	4.93 (1.68) [1.00–9.00]
	M+	Correct	5.22 (1.47) [2.50–9.00]	5.49 (1.40) [2.67–9.00]	5.04 (1.64) [1.58–9.00]

Table A3.2. *Participant-level descriptive statistics for enjoyment, confidence, and arousal ratings categorised by congruency and memory on Day 2. Values are means, with standard deviations in parentheses and ranges in square brackets.*

			**Enjoyment**	**Confidence**	**Tiredness**
Reading	M-	Incorrect	4.70 (1.34) [1.00–7.00]	4.36 (1.51) [1.00–7.00]	5.55 (1.38) [2.33–8.00]
	M-	Correct	4.58 (1.32) [1.00–7.00]	4.37 (1.38) [1.00–7.67]	5.47 (1.32) [2.25–8.00]
	M+	Incorrect	5.13 (1.28) [1.25–7.44]	5.33 (1.37) [2.00–8.00]	5.61 (1.22) [2.50–8.00]
	M+	Correct	5.46 (1.07) [2.60–8.38]	5.60 (1.04) [2.60–7.33]	5.71 (1.28) [2.00–8.00]
Reading and Listening	M-	Incorrect	5.10 (1.64) [1.00–8.50]	4.73 (1.72) [1.00–7.75]	5.67 (1.52) [2.00–9.00]
	M-	Correct	5.15 (1.63) [2.00–8.40]	4.68 (1.94) [1.00–8.50]	5.69 (1.48) [2.00–9.00]
	M+	Incorrect	5.60 (1.24) [3.00–8.20]	5.41 (1.49) [2.50–8.00]	5.66 (1.47) [2.70–9.00]
	M+	Correct	5.55 (1.48) [2.00–8.14]	5.67 (1.67) [2.33–9.00]	5.54 (1.34) [3.00–9.00]
Listening	M-	Incorrect	4.21 (1.19) [1.00–6.00]	4.00 (1.40) [1.00–7.00]	4.83 (1.40) [1.75–8.25]
	M-	Correct	4.58 (1.70) [1.33–9.00]	4.11 (1.75) [1.67–9.00]	4.79 (1.80) [1.33–9.00]
	M+	Incorrect	5.08 (1.65) [2.43–9.00]	5.30 (1.56) [2.60–9.00]	5.04 (1.80) [1.43–9.00]
	M+	Correct	5.46 (1.39) [2.60–9.00]	5.75 (1.25) [3.33–9.00]	5.11 (1.61) [1.80–9.00]
