# Supplementary material for: Intrinsic Reward Modulates Word Learning in Both Oral and Written Contexts
Source: J Cogn. 2026 Apr 30;9(1):28. doi: 10.5334/joc.499 (PMC13131340; doi:10.5334/joc.499)
Supplement: Appendix 4. — Memory analyses controlling for delay time. [file joc-9-1-499-s4.pdf]

Appendix 4. Memory analyses controlling for delay time

To examine whether variation in the delay between the learning and memory phases influenced the results, we conducted an exploratory analysis excluding participants with long delays. We retained data from 92 participants who completed the memory phase within 38 hours of learning (Reading: n = 30; Reading and Listening: n = 31; Listening: n = 31).

As in the full sample, the final model fitted to the enjoyment data (glmer(Enjoyment ~ Congruency + Memory + Modality + Congruency:Memory + (1 | Participant) + (1 | Item))) indicated that both congruency and memory significantly predicted enjoyment (see Table A4.1). Crucially, the predicted interaction between congruency and memory remained significant. As shown in Figure A4.1, the pattern of results closely replicated that observed in the full dataset.

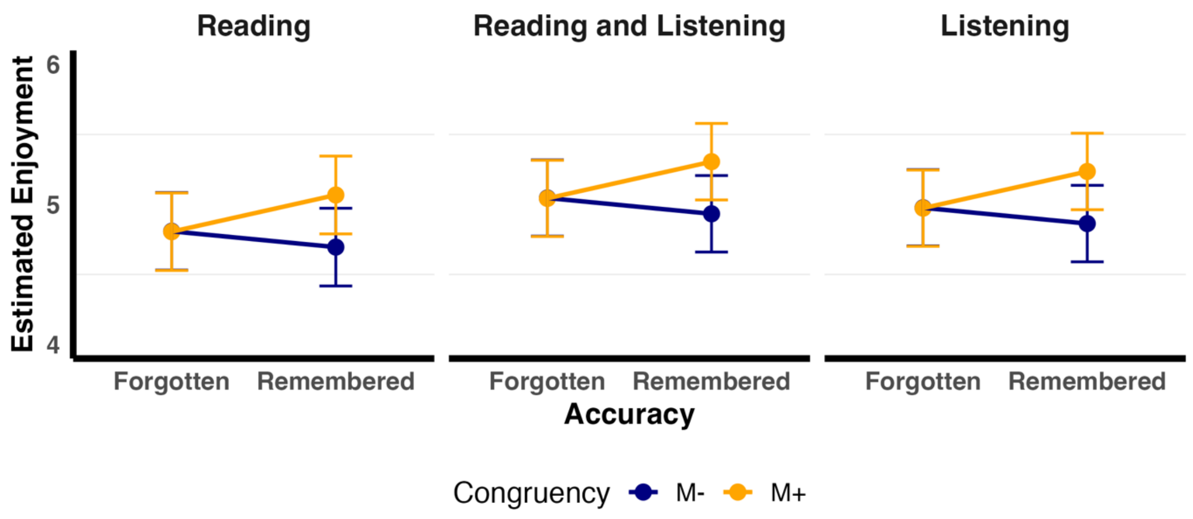

Figure A4.1. *Estimated marginal means of enjoyment by Accuracy, Congruency and Modality. The figure displays the estimated marginal means (EMMs) of enjoyment as a function of Memory*

("Incorrect" vs. "Correct") and Congruency (M- vs. M+), with separate panels for each Modality. Error bars show ±1 standard error (SE).

Table A4.1. *Generalized Linear Mixed-Effects Results for Enjoyment using Day 2 Memory in participants with memory responses under 12 hours*

**Measure**	**Fixed effect**		**SE**	**95% CI**	**z-value**
Enjoyment					
	(Intercept)	.84	0.28	4.30, 5.39	**17.60**
	**Congruency**	0.09	0.02	-0.13, -0.06	**-5.31**
	**Memory**	0.04	0.02	-0.07, 0.00	**-2.09**
	Modality (Reading and Listening)	.24	0.39	-0.53, 1.00	0.62
	Modality (Listening)	.17	0.39	-0.60, 0.93	0.44
	**Congruency * Memory**	.09	0.02	0.06, 0.13	**5.15**
**Random effects**			**Variance**	**SD**	
Participant	(intercept)		2.241	1.497	
Word	(intercept)		0.011	0.104	
