# Supplementary material for: Intrinsic Reward Modulates Word Learning in Both Oral and Written Contexts
Source: J Cogn. 2026 Apr 30;9(1):28. doi: 10.5334/joc.499 (PMC13131340; doi:10.5334/joc.499)
Supplement: Appendix 5. — The effect on arousal ratings. [file joc-9-1-499-s5.pdf]

Appendix 5. The effect on arousal ratings

Testing hypothesis 1: Arousal ratings will be higher when word meanings are successfully extracted across modalities.

The final model fitted to Arousal data (glmer(Arousal ~ Congruency + Accuracy + Modality + Congruency:Accuracy + (1 | Participant) + (1 | Item)) indicated that accuracy significantly predicted arousal (see Table A5.1). Accurate trials were associated with greater arousal than inaccurate trials. Participants also reported lower arousal in the listening condition, relative to the reading and reading and listening condition. Surprisingly, we also observed a significant two-way interaction between congruency and accuracy. Follow up analyses showed this was driven by higher arousal when successfully inferring the meaning of a word relative to unsuccessful inference (z=3.98,p=.0004), and also relative to correct rejection (z=3.02,p=.013). Further, these effects were much smaller than in enjoyment.

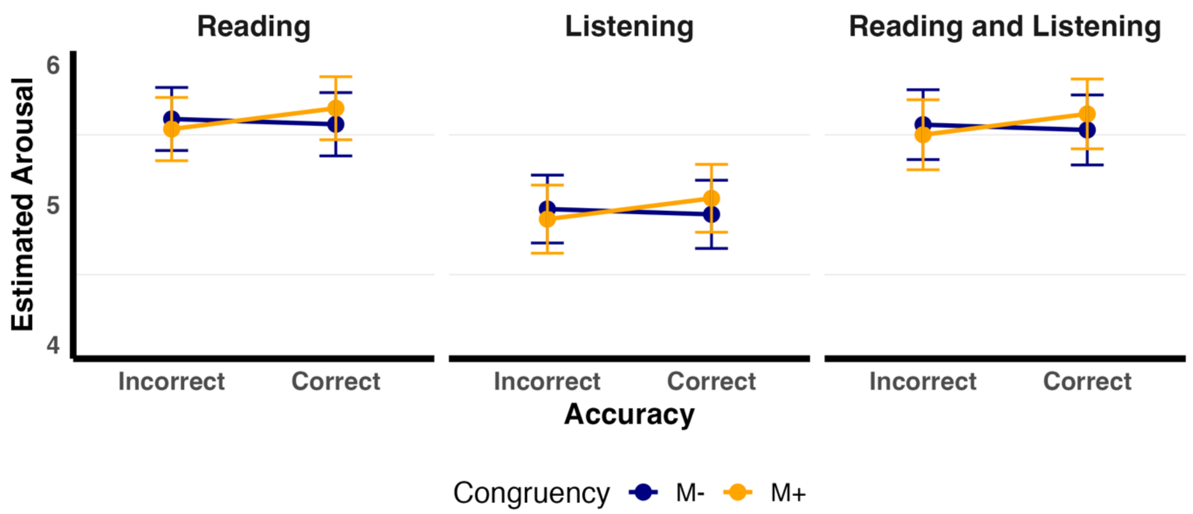

Figure A5.1. Estimated marginal means of Arousal by Accuracy, Congruency and Modality. The figure displays the estimated marginal means (EMMs) of enjoyment as a function of Accuracy ("Incorrect" vs. "Correct") and Congruency (M- vs. M+), with separate panels for each Modality. Error bars show ±1 standard error (SE).

Table A5.1. Generalized Linear Mixed-Effects Results for Arousal using Day 1 Accuracy

**Measure**	**Fixed effect**	**b**	**SE**	**95% CI**	**z-value**
Enjoyment	(Intercept)	5.61	0.23	5.16, 6.05	**25.00**
	Congruency	-0.01	0.01	-0.04,0.01	-0.83
	Accuracy	-0.03	0.01	-0.05,0.00	**-2.07**
	Modality (Reading and Listening)	-0.04	0.33	-0.70,0.61	-0.12
	Modality (Listening)	-0.64	0.33	-1.29, 0.00	**-1.98**
	Congruency * Accuracy	0.05	0.01	0.02,0.07	**3.43**
**Random effects**			**Variance**	**SD**	
Participant	(intercept)		2.03	1.43	
Word	(intercept)		0.06	0.25	

Testing hypothesis 2: Memory for word meanings will be predicted by arousal across modalities

The final model fitted to Arousal data (glmer(Arousal ~ Congruency + Memory + Modality + Congruency: Modality + Congruency: Memory + Congruency: Modality: Memory + (1 | Participant) + (1 | Item)) indicated that arousal differed across modality (see Table A5.2, Figure A5.2). Participant showed lowered arousal in the listening condition, and this was also modulated by memory. Critically, there was no significant interaction between congruency and modality on arousal.

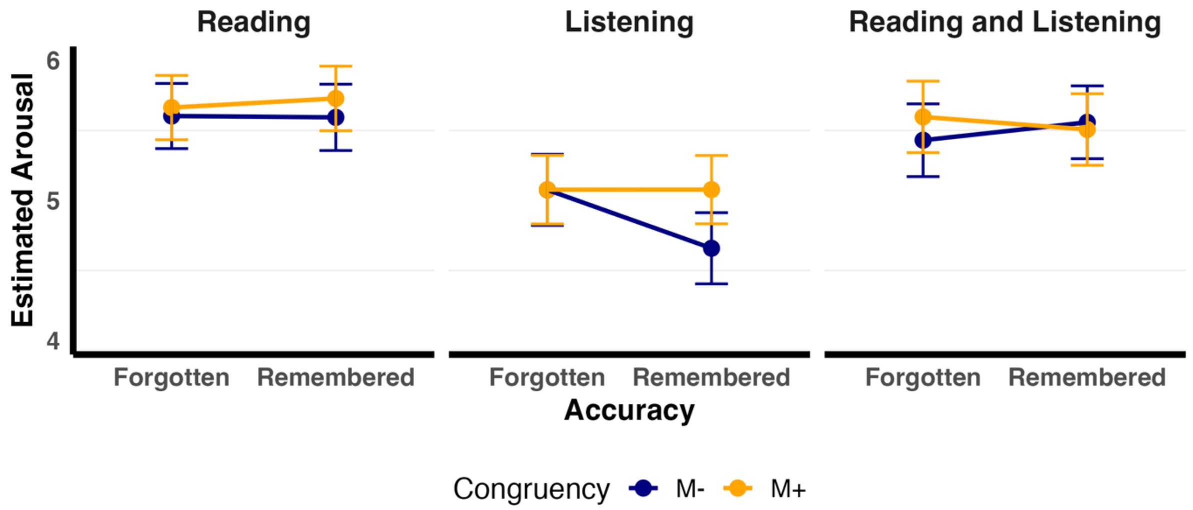

Figure A5.2. Estimated marginal means of Arousal by Memory, Congruency and Modality. The figure displays the estimated marginal means (EMMs) of arousal as a function of Memory ("Forgotten" vs. "Remembered") and Congruency (M- vs. M+), with separate panels for each Modality. Error bars show ±1 standard error (SE).

Table A5.2. Generalized Linear Mixed-Effects Results for Arousal using Day 2 Memory

**Measure**	**Fixed effect**	**b**	**SE**	**95% CI**	**z-value**
Enjoyment	(Intercept)	5.65	0.23	5.20,6.09	**25.00**
	Congruency	-0.05	0.03	-0.11,0.02	-1.46
	Memory	-0.01	0.03	-0.08,0.05	-0.44
	Modality (Listening)	-0.68	0.32	-1.32,-0.03	**-2.08**
	Modality (Reading and Listening)	-0.12	0.33	-0.78,0.53	-0.37
	Memory: Modality (L)	0.12	0.05	0.02,0.21	**2.45**
	Memory: Modality (RL)	0.00	0.05	-0.09,0.09	0.09
	Congruency: Modality (L)	-0.06	0.05	-0.15,0.04	-1.13
	Congruency: Modality (RL)	0.02	0.05	-0.07,0.11	0.42
	Congruency: Memory	0.02	0.03	-0.04,0.08	0.58
	Congruency: Memory: Modality (L)	0.09	0.05	-0.01,0.18	1.77
	Congruency: Memory: Modality (RL)	-0.07	0.05	-0.16,0.02	-1.59
**Random effects**			**Variance**	**SD**	
Participant	(intercept)		1.97	1.40	
Word	(intercept)		0.08	0.27	
